# Downregulation of CR6-interacting factor 1 suppresses keloid fibroblast growth via the TGF-β/Smad signaling pathway

**DOI:** 10.1038/s41598-020-79785-y

**Published:** 2021-01-12

**Authors:** Harsha Nagar, Sungmin Kim, Ikjun Lee, Seonhee Kim, Su-Jeong Choi, Shuyu Piao, Byeong Hwa Jeon, Sang-Ha Oh, Cuk-Seong Kim

**Affiliations:** 1 Department of Medical Science, Chungnam National University, Daejeon, Republic of Korea; 2 Department of Physiology, School of Medicine, Chungnam National University, 55 Munhwa-ro, Jung-Gu, Daejeon, 301-131 Republic of Korea; 3 Department of BK21 Plus CNU Integrative Biomedical Education Initiative, Chungnam National University, Daejeon, Republic of Korea; 4 Department of Plastic and Reconstructive Surgery, School of Medicine, Chungnam National University, 282 Munhwa-ro, Jung-Gu, Daejeon, 35015 Republic of Korea; 5 Brain Research Institute, School of Medicine, Chungnam National University, Daejeon, Republic of Korea

**Keywords:** Physiology, Diseases, Medical research

## Abstract

Keloids are a type of aberrant skin scarring characterized by excessive accumulation of collagen and extracellular matrix (ECM), arising from uncontrolled wound healing responses. While typically non-pathogenic, keloids are occasionally regarded as a form of benign tumor. CR6-interacting factor 1 (CRIF1) is a well-known CR6/GADD45-interacting protein, that has both nuclear and mitochondrial functions, and also exerts regulatory effects on cell growth and apoptosis. In this study, cell proliferation, cell migration, collagen production and TGF-β signaling was compared between normal fibroblasts (NFs) and keloid fibroblasts (KFs). Subsequently, the effects of CRIF1 deficiency were investigated in both NFs and KFs. Cell proliferation, cell migration, collagen production and protein expressions of TGF-β, phosphorylation of Smad2 and Smad3 were all found to be higher in KFs compared to NFs. CRIF1 deficiency in NFs and KFs inhibited cell proliferation, migration, and collagen production. In addition, phosphorylation of Smad2 and Smad3, which are transcription factors of collagen, was decreased. In contrast, mRNA expression levels of Smad7 and SMURF2, two important inhibitory proteins of Smad2/3, were increased, suggesting that CRIF1 may regulate collagen production. CRIF1 deficiency decreases the proliferation and migration of KFs, thereby inhibiting their overgrowth via the transforming growth factor-β (TGF-β)/Smad pathway. CRIF1 may therefore represent a potential therapeutic target in keloid pathogenesis.

## Introduction

Keloid disease, or keloid scarring, is a condition in which pathological scar tissue grows aggressively beyond the boundary of the original wound. These scars subsequently invade the surrounding healthy skin, leading to pain, itching, and a stretching sensation^1^. The growth of keloids is unique to humans, and there is no evidence of malignant potential. The primary driver of keloid formation is an abnormal elevation of fibroblast activity, leading to a greater amount of connective tissue proliferation and hyaline degeneration^2^. While this abnormal increase in fibroblast activity is a key characteristic of keloid development, the pathophysiological mechanisms underlying keloid formation have yet to be elucidated. Although surgical excision is effective, the recurrence rate is high, potentially leading to an even more severe condition^3^.

The transforming growth factor-β (TGF-β)/Smad signaling pathway is important in the excessive proliferation of fibroblasts and collagen accumulation seen in keloid pathogenesis^4,5^. Keloid fibroblast (KF)-mediated inflammation is essential to disease pathology, and is driven by the abnormal secretion of proinflammatory mediators along with an abnormal response to other inflammatory signals^6^. Numerous studies have shown that KFs exhibit increased expression of many potent cytokines, including TGF-β^7,8^ relative to normal fibroblasts (NFs). In addition to increased cytokine production, KFs also exhibit increased transcription of numerous cytokine receptors relative to NFs, leading to excessive synthesis of proteoglycans, collagen, and other extracellular matrix (ECM) components.

ECM is made of collagen and elastin fibers dispersed in ground substance, a complex mixture of glycosaminoglycans, proteoglycans, and connective tissue glycoproteins. ECM is able to modulate wound repair, either directly by modulation of important aspects of cell behavior, such as migration, adhesion, proliferation, and survival, or indirectly by modulation of extracellular protease secretion, growth factor activity, and bioavailability. Keloids exhibit increased levels of acid-soluble collagen, proteoglycans, and water relative to both normal and hypertrophic scars^9^. Taken together, these findings indicate that keloids can be characterized in terms of overgrowth of fibroblasts, migration outside of the normal wound region, and excessive synthesis of ECM.

CR6-interacting factor 1 (CRIF1) is ubiquitously expressed in humans. It interacts with three members of the Gadd 45 family of proteins, Gadd45α, Gadd45β, Gadd45γ, as well as the orphan nuclear receptor Nur77. Under normal conditions, CRIF1 is primarily localized to the nucleus, where it colocalizes with Gadd45γ^10^. In the nucleus, its primary function is as a regulator of cell cycle progression and cell growth^11^; however, it also acts as a transcriptional coactivator of STAT3, influencing a variety of biological functions including transcription, DNA binding, and cellular transformation^12^. As a result, CRIF1 knockout embryos show lethality and developmental arrest, along with defective proliferation and severe apoptosis. CRIF1 is also found in the mitochondria, where it is associated with large mitoribosomal subunits and is responsible for the production of oxidative phosphorylation (OXPHOS) polypeptides and their subsequent insertion into the inner mitochondrial membrane^13^. Accordingly, both CRIF1 knockout mice and CRIF1 downregulation in vitro exhibit features of mitochondrial dysfunction^14^. Currently, little is known regarding the pathogenesis, treatment, and prevention of recurrent keloids. Biosynthetic metabolism of keloids has been shown to exhibit features similar to cancer metabolism^15^. Furthermore, induction of mitochondrial dysfunction is emerging as a new therapeutic strategy for treatment of tumor cells.

The aim of our study was to downregulate CRIF1 expression in NFs and KFs, to assess its effects on cell proliferation, migration, and collagen production. We suggest that suppression of CRIF1 may be an important factor in keloid treatment.

## Results

### Cell proliferation is higher in keloid fibroblasts

**Figure 1:**
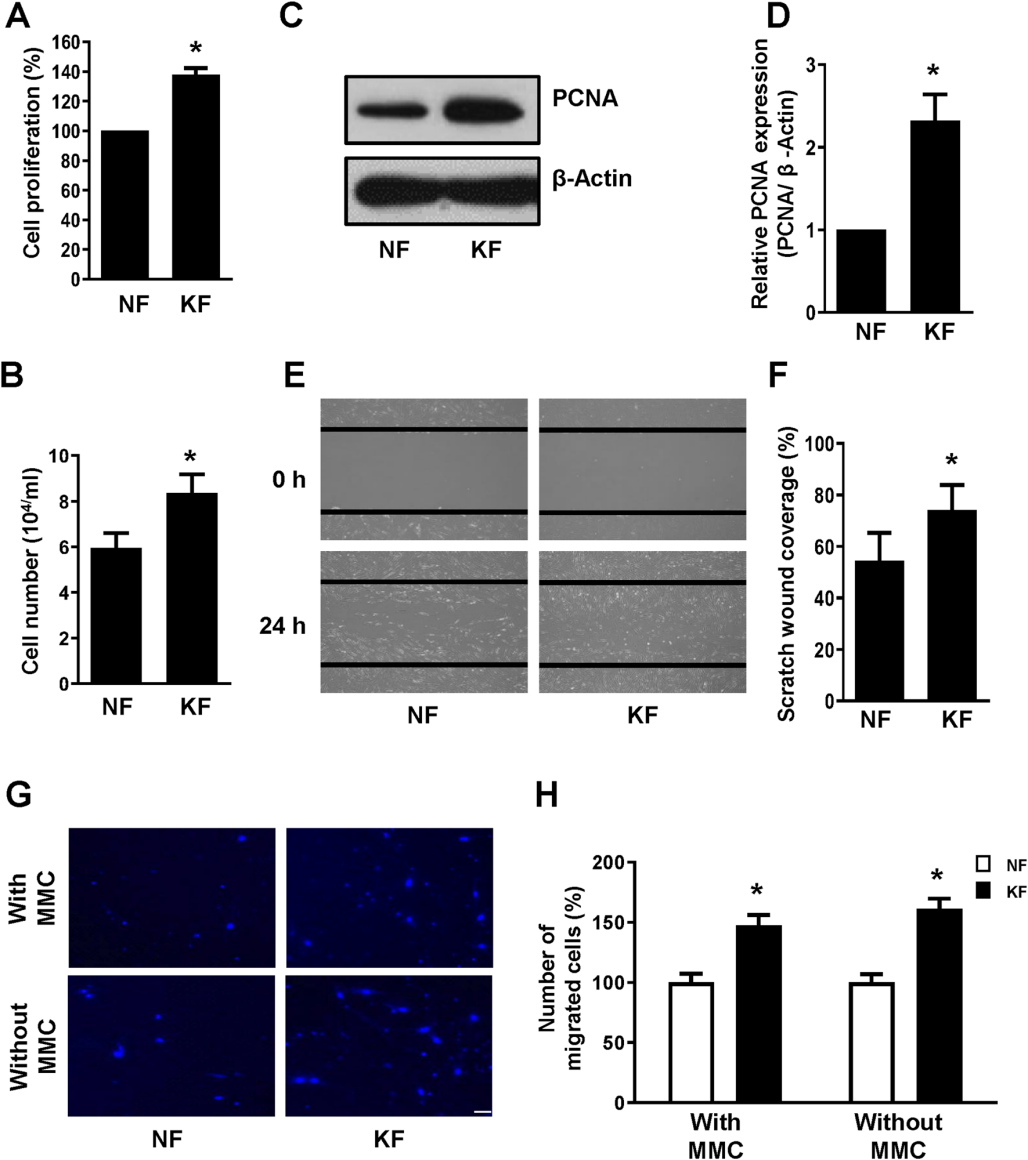
Comparison of cell proliferation and cell migration between normal fibroblasts (NFs) and keloid fibroblasts (KFs). (**A**) NFs and KFs were cultured in 6-well plates for 24 h. After incubation, cells were treated with Cell Counting Kit-8 (CCK-8) solution, with cell proliferation measured based on absorbance at 450 nm. (**B**) NFs and KFs were cultured in 6-well plates for 24 h. After incubation, cells were harvested and ADAM-MC kit was used for cell counting. (**C**) Proliferating cell nuclear antigen **(**PCNA) protein expression, used as a marker of cell proliferation, was measured by western blot; β-actin was used as the loading control. Full-length blots are presented in Supplementary Fig. S1. (**D**) PCNA density was calculated using ImageJ software. (**E**) NFs and KFs were cultured in 6-well plates for 24 h followed by wounding for 24 h. Images were taken using a light microscope. (**F**) Quantification of wound closure was performed using ImageJ software. (**G**) Transwell assay was conducted to determine cell migration with or without MMC treatment. Scale bar 200 μm. (H) Quantification of the number of migrated cells was performed using ImageJ software. All data are presented as the mean ± SD of three independent experiments. *P < 0.05 relative to NFs.

One of the most important characteristics of keloids is uncontrolled fibroblast proliferation. First, we measured the difference in cell proliferation between NFs and KFs using CCK-8 and ADAM-MC automatic cell counter. All fibroblasts were obtained by primary cell culture of human skin samples. Cell proliferation results demonstrated that the proliferation of KFs was significantly higher than that of NFs (Fig. 1A,B).

Proliferating cell nuclear antigen (PCNA) is a DNA clamp. Given that it is essential for DNA replication, PCNA is often used as a marker of cell proliferation. PCNA protein expression levels in KFs were significantly higher than in NF, as evidenced by Western blot (Fig. 1C,D).

### Cell migration is higher in keloid fibroblasts

Next, we performed a cell scratch assay to assess differences in cell migration between NFs and KFs. Cells were seeded in 6-well plates and incubated for 24 h, after which a scratch was made through the center of each well. Photographs were taken at baseline and 24 h after scratching. Under basal conditions, in the absence of any exogenous modulation, the number of cells covering the wound site was significantly higher in KFs at 24 h after injury, as compared to NFs (Fig. 1E,F).

We also performed transwell assay to detect cell migration. Cells were seeded in 6-well plates and incubated for 24 h, after which they were transferred to the upper transwell chamber in serum free medium. The lower chamber contained complete medium. After 24 h of incubation, the migrated cells were stained with DAPI and counted under a microscope. To rule out cell proliferation as the cause of migration in these experiments; cells were treated with a cell proliferation inhibitor Mitomycin C. As shown in Fig. 1G,H, mitomycin C treatment slowed down fibroblast proliferation as seen by a reduction in the total cell number (data not shown), but it did not cause complete cell death to affect cell migration and therefore the graph shows that the pattern of cell migration was similar in both the conditions (with or without Mitomycin C treatment). Hence, it was confirmed that migration of KFs was higher than NFs.

### The TGF-β/Smad signaling pathway is activated in keloid fibroblasts

**Figure 2:**
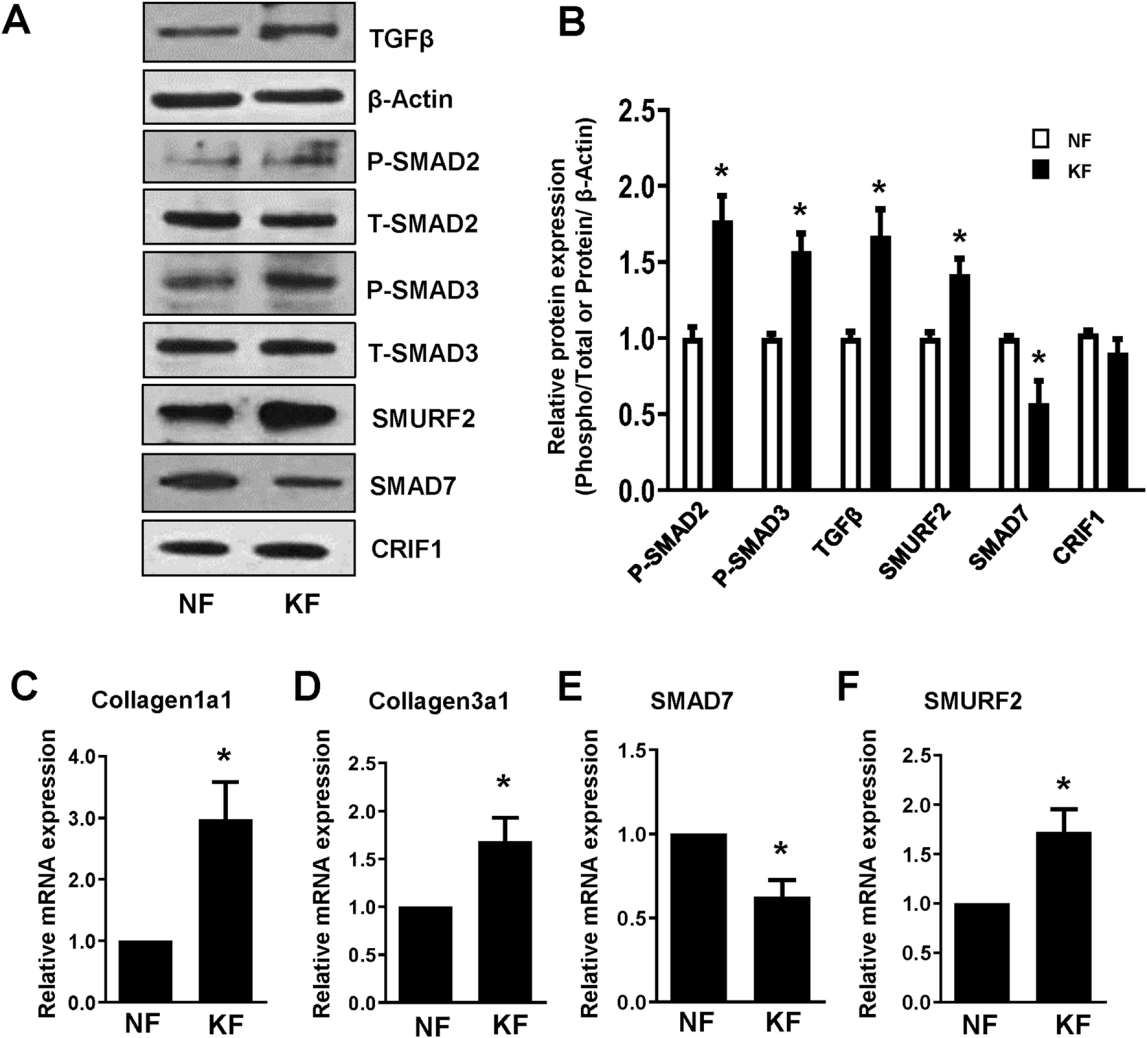
Comparison of transforming growth factor-β (TGF-β)/Smad expression levels between normal fibroblasts (NFs) and keloid fibroblasts (KFs). (**A**) NFs and KFs were cultured in 6-well plates for 24 h. After 24 h incubation, cells were harvested, lysed, and analyzed by western blot to assess differences in protein levels of various proteins of the TGF-β/Smad signaling pathway and CRIF1; β-actin was used as the loading control. Full-length blots are presented in Supplementary Fig. S2. (**B**) Protein density was calculated using ImageJ software. The mRNA levels of (**C**) collagen 1A1 (COL1A1), (**D**) collagen 3A1 (COL3A1) (**E**) Smad7 and (**F**) SMURF2 were determined by quantitative polymerase chain reaction (qPCR) in NFs and KFs. All data are presented as the mean ± SD of three independent experiments. *P < 0.05 relative to NFs.

Among the many growth factors involved in the wound healing process, TGF-β appears to play a central role. TGF-β protein expression levels were found to be significantly higher in KFs than NFs in this study (Fig. 2A,B). The TGF-β/Smad signaling pathway plays an important role in keloid formation. Smad proteins are signaling proteins downstream of TGF-β that mediate the intracellular signaling transduction of TGF-β. TGF-β binds to high-affinity receptors that phosphorylate and activate the downstream substrates Smad2 and Smad3. Protein expression levels of phosphorylated Smad2 and Smad3, two major transcription factors of ECM, were found to be higher in KFs than NFs (Fig. 2A,B).

Furthermore, Inhibitory Smads (Smad6 and Smad7) are antagonists for the activity of receptor-regulated Smads, leading to the termination of TGF-β signaling. Between these two proteins, Smad7 is more specific for TGF-β signaling, with lower protein expression of SMAD7 in KFs as compared to NFs (Fig. 2A,B). Smad ubiquitination regulatory factor 2 (SMURF2), a Smad-specific ubiquitin ligase, also plays a critical role in the regulation of TGF-β/Smad signaling. Here, we found that the protein expression level of SMURF2 was higher in KFs than in NFs. At the same time, no significant difference was found in CRIF1 protein expression at the basal level between NFs and KFs (Fig. 2A,B).

### Expression of ECM components is upregulated in keloid fibroblasts

A tightly regulated balance between the synthesis and degradation of ECM is essential for normal scar formation. However, if this balance shifts towards increased ECM production or decreased degradation, keloids or hypertrophic scars may emerge. To compare the ECM synthesis of NFs and KFs in our study, mRNA expression levels of the ECM components collagen 1A1 (COL1A1) and collagen 3A1 (COL3A1) were measured by quantitative PCR (qPCR). The mRNA expression levels of both COL1A1 and COL3A1 were higher in KFs than NFs (Fig. 2C,D). At the same time, the mRNA expression of SMAD7 was found to be lower in KFs whereas, SMURF2 mRNA expression was higher in KFs as compared to NFs (Fig. 2E,F).

### Cell proliferation is altered by CRIF1 downregulation in normal and keloid fibroblasts

**Figure 3:**
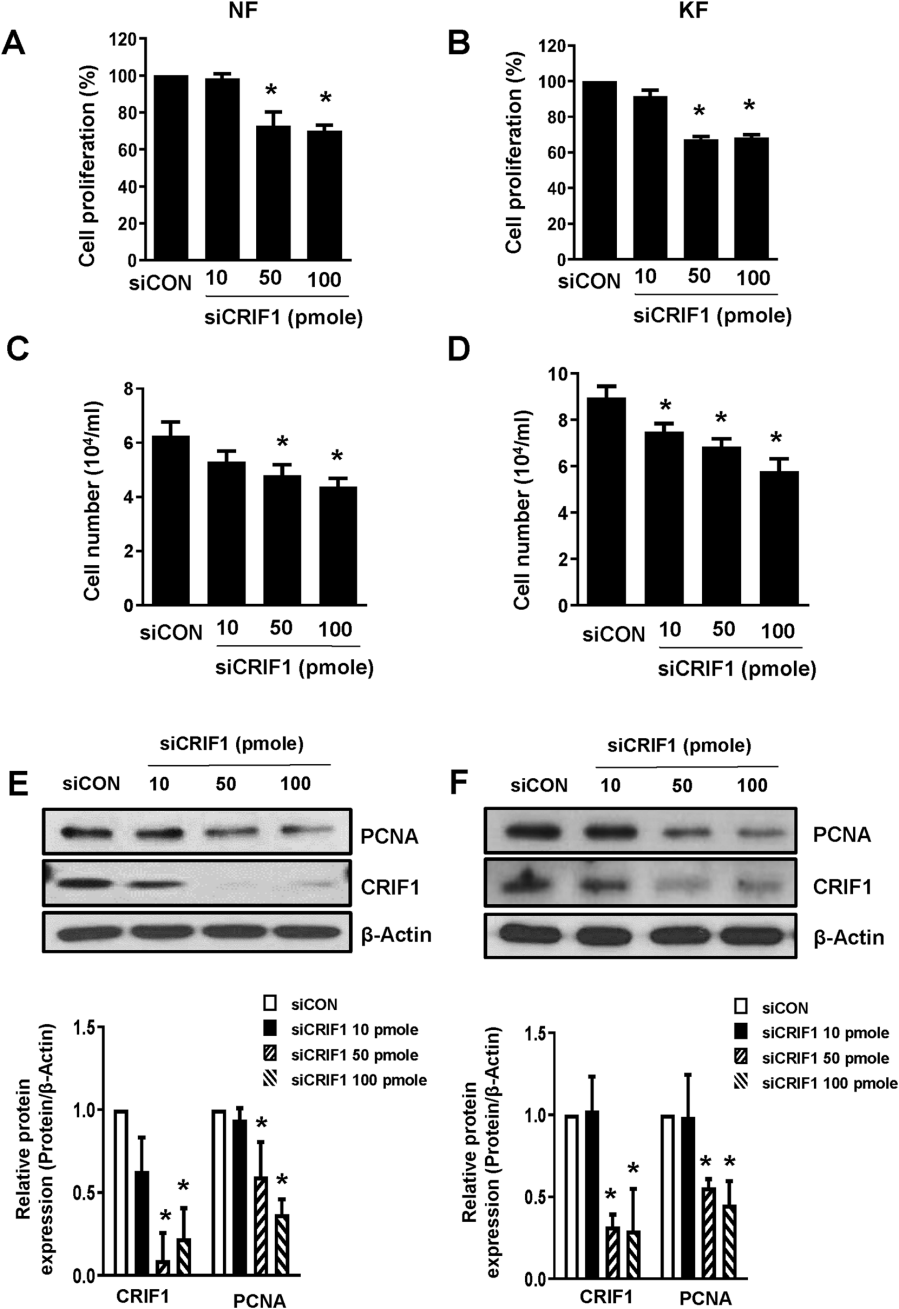
CR6-interacting factor 1 (CRIF1) downregulation leads to decreased cell proliferation in normal fibroblasts (NFs) and keloid fibroblasts (KFs). NFs were transfected with CRIF1 small interfering RNA (siRNA) in a dose-dependent manner for 48 h. (**A**) After incubation, cells were treated with CCK-8 solution, with cell proliferation measured based on absorbance at 450 nm. (**C**) After incubation, cells were harvested and ADAM-MC kit was used for cell counting. (**E**) PCNA protein levels were measured by Western blot; β-actin was used as the loading control. PCNA density was calculated using ImageJ software. Full-length blots are presented in Supplementary Fig. S3. KFs were transfected with CRIF1 small interfering RNA (siRNA) in a dose-dependent manner for 48 h. (**B**) After incubation, cells were treated with CCK-8 solution, with cell proliferation measured based on absorbance at 450 nm. (**D**) After incubation, cells were harvested and ADAM-MC kit was used for cell counting. (**F**) PCNA protein levels were measured by western blot; β-actin was used as the loading control. PCNA density was calculated using ImageJ software. Full-length blots are presented in Supplementary Fig. S4. All data are presented as means ± SD of three independent experiments. *P < 0.05 compared to siCON.

Cell proliferation is a rapid increase in cell number, which is influenced by various growth factors. At present, there are numerous ongoing studies on keloid, but the specific molecular biological mechanism of proliferation and invasion of keloids has not been successfully elucidated. Nevertheless, it is well known that KFs have a reduced growth factor requirement for proliferation^16^ and that keloids are composed of fibrous tissue which has a high proliferation rate as compared with that of NFs^17–19^. To examine the effects of CRIF1 downregulation on fibroblast cell proliferation, we transfected NFs and KFs with control and dose-dependent CRIF1 siRNA. After 48 h of transfection, cell proliferation was measured using CCK-8 assay and ADAM-MC automatic cell counter. We observed that the proliferation of both NFs (Fig. 3A,C) and KFs (Fig. 3B,D) transfected with CRIF1 siRNA was significantly reduced compared with the control cells. At the same time, PCNA protein levels were also reduced in NFs (Fig. 3E) and KFs (Fig. 3F) in response to CRIF1 downregulation.

### Cell migration is altered by CRIF1 downregulation in normal and keloid fibroblasts

**Figure 4:**
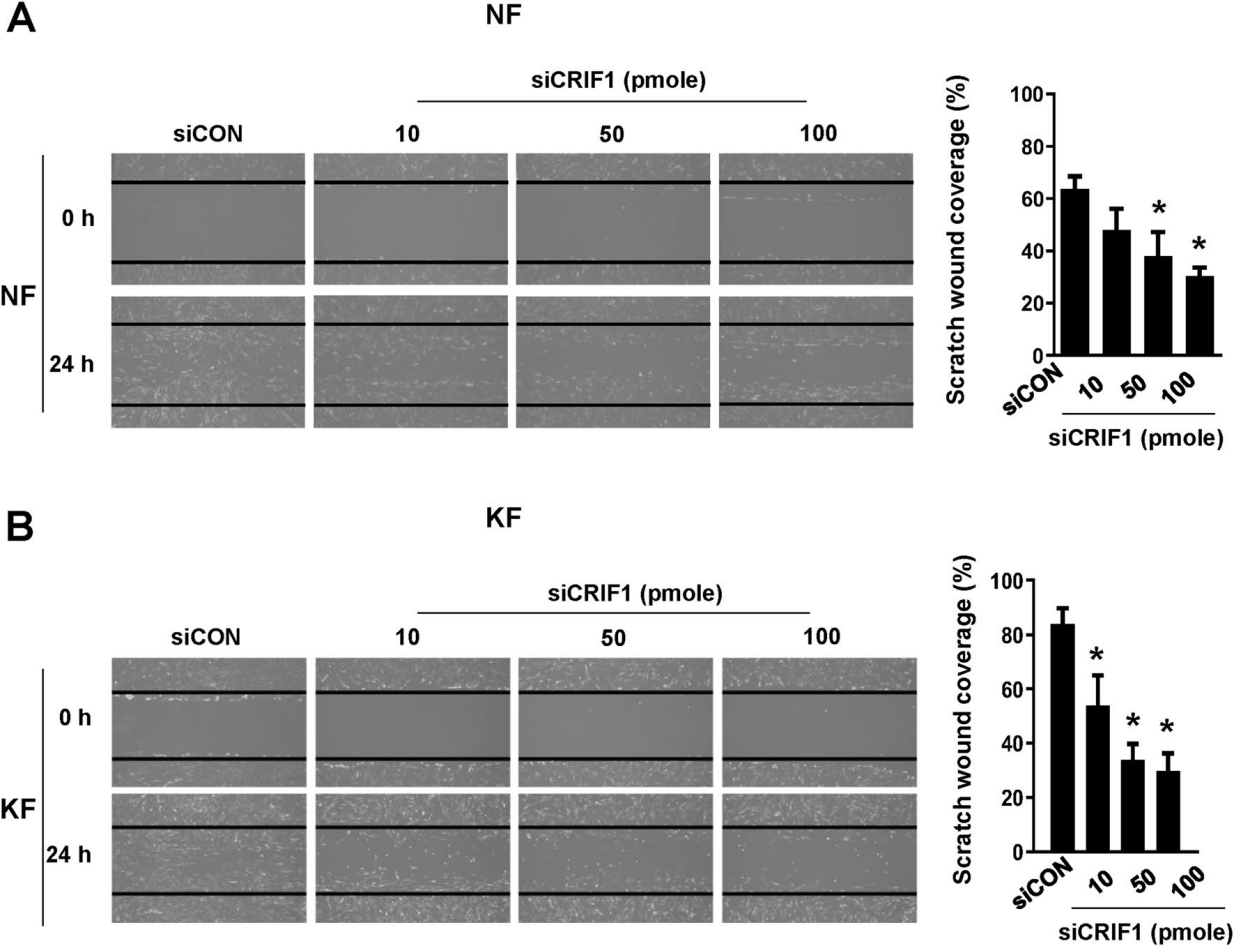
CR6-interacting factor 1 (CRIF1) downregulation leads to decreased cell migration measured by scratch wound healing assay in normal fibroblasts (NFs) and keloid fibroblasts (KFs). (**A**) NFs were transfected with CRIF1 siRNA in a dose-dependent manner for 24 h, followed by wounding for 24 h. (**B**) KFs were transfected with CRIF1 siRNA in a dose-dependent manner for 24 h, followed by wounding for 24 h. Images were taken using a light microscope. Quantification of wound closure was performed using ImageJ software. All data are presented as means ± SD of three independent experiments. *P < 0.05 compared to siCON.

Cell migration is a central process of wound healing and is also important for keloid formation. As shown in Fig. 1, KFs migrate at a faster rate as compared to NFs. In addition, KFs show changed responses to numerous growth factors that influence multiple processes including cell migration as well as cell proliferation^20^. Cell migration of NFs and KFs after CRIF1 downregulation was measured using a cell scratch assay. Cells transfected with control or CRIF1 siRNA were incubated for 24 h, after which a scratch was made through the center of each well. Photographs were taken at baseline and 24 h after scratching. NFs (Fig. 4A) and KFs (Fig. 4B) migrated into the wound area and nearly covered the whole scratch after 24 h, while cells transfected with CRIF1 siRNA did not migrate as far suggesting that CRIF1 downregulation significantly reduced cell migration.

**Figure 5:**
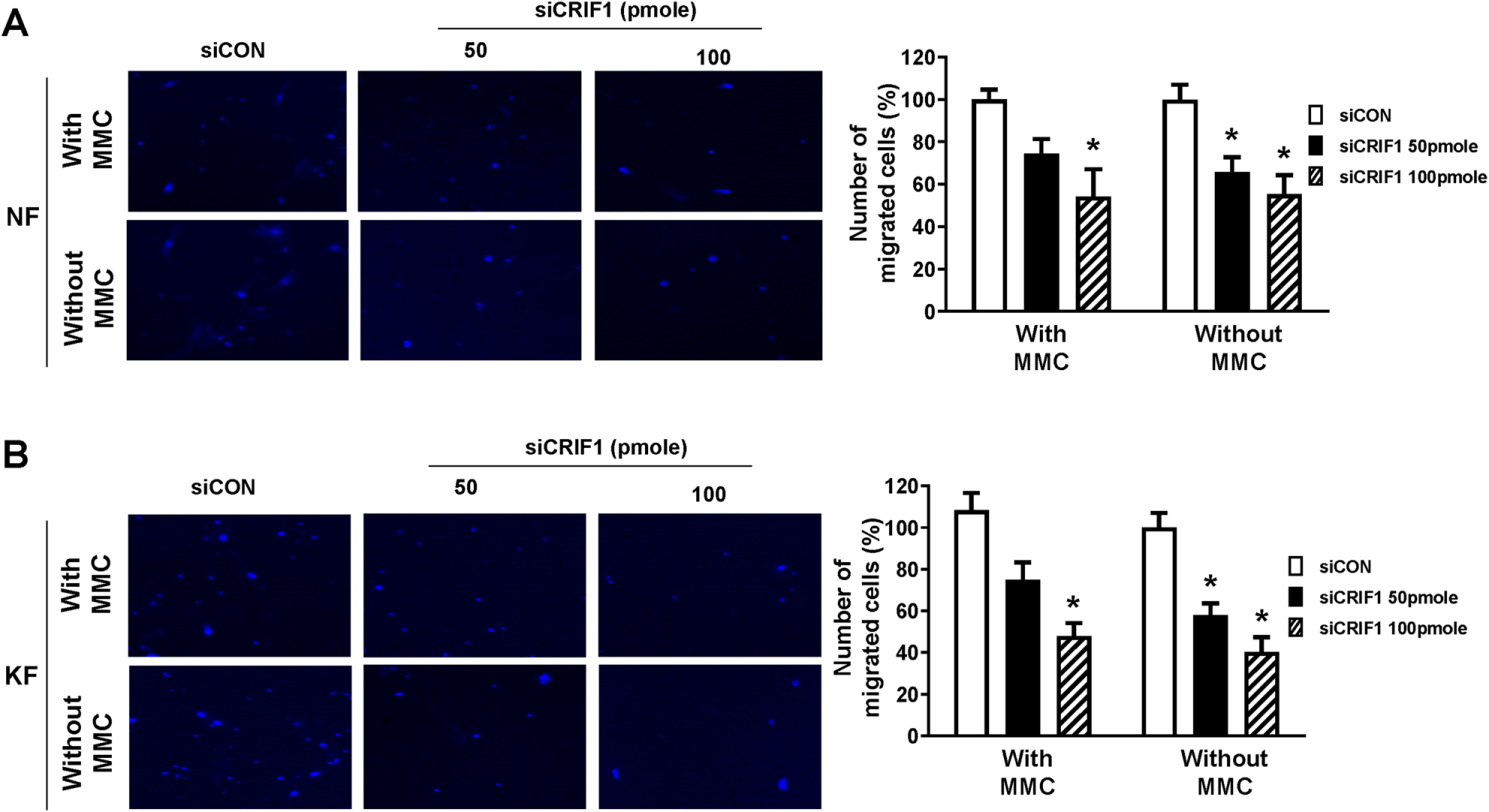
CR6-interacting factor 1 (CRIF1) downregulation leads to decreased cell migration measured by transwell assay in normal fibroblasts (NFs) and keloid fibroblasts (KFs). (**A**) NFs were transfected with CRIF1 siRNA in a dose-dependent manner and transwell assay was conducted to determine cell migration with or without MMC treatment. Scale bar 200 μm. (**B**) KFs were transfected with CRIF1 siRNA in a dose-dependent manner and transwell assay was conducted to determine cell migration with or without MMC treatment. Scale bar 200 μm. Quantification of migrated cells was performed using ImageJ software. All data are presented as means ± SD of three independent experiments. *P < 0.05 compared to siCON.

Cell migration was also measured by the transwell assay. Cells transfected with control or CRIF1 siRNA were incubated for 24 h, after which they were transferred to the upper transwell chamber in serum free medium. The lower chamber contained complete medium. After 24 h of incubation, the migrated cells were stained with DAPI and counted under a microscope. To rule out cell proliferation as the cause of migration in these experiments; cells were treated with a cell proliferation inhibitor Mitomycin C. As shown in Fig. 5A,B, mitomycin C treatment slowed down fibroblast proliferation as seen by a reduction in the total cell number (data not shown), but it did not cause complete cell death to affect cell migration and therefore the graph shows that the pattern of cell migration was similar in both the conditions (with or without Mitomycin C treatment). It was confirmed that migration of NFs (Fig. 5A) and KFs (Fig. 5B) was reduced by CRIF1 downregulation.

### The TGF-β/Smad signaling pathway is altered by CRIF1 downregulation in normal and keloid fibroblasts

**Figure 6:**
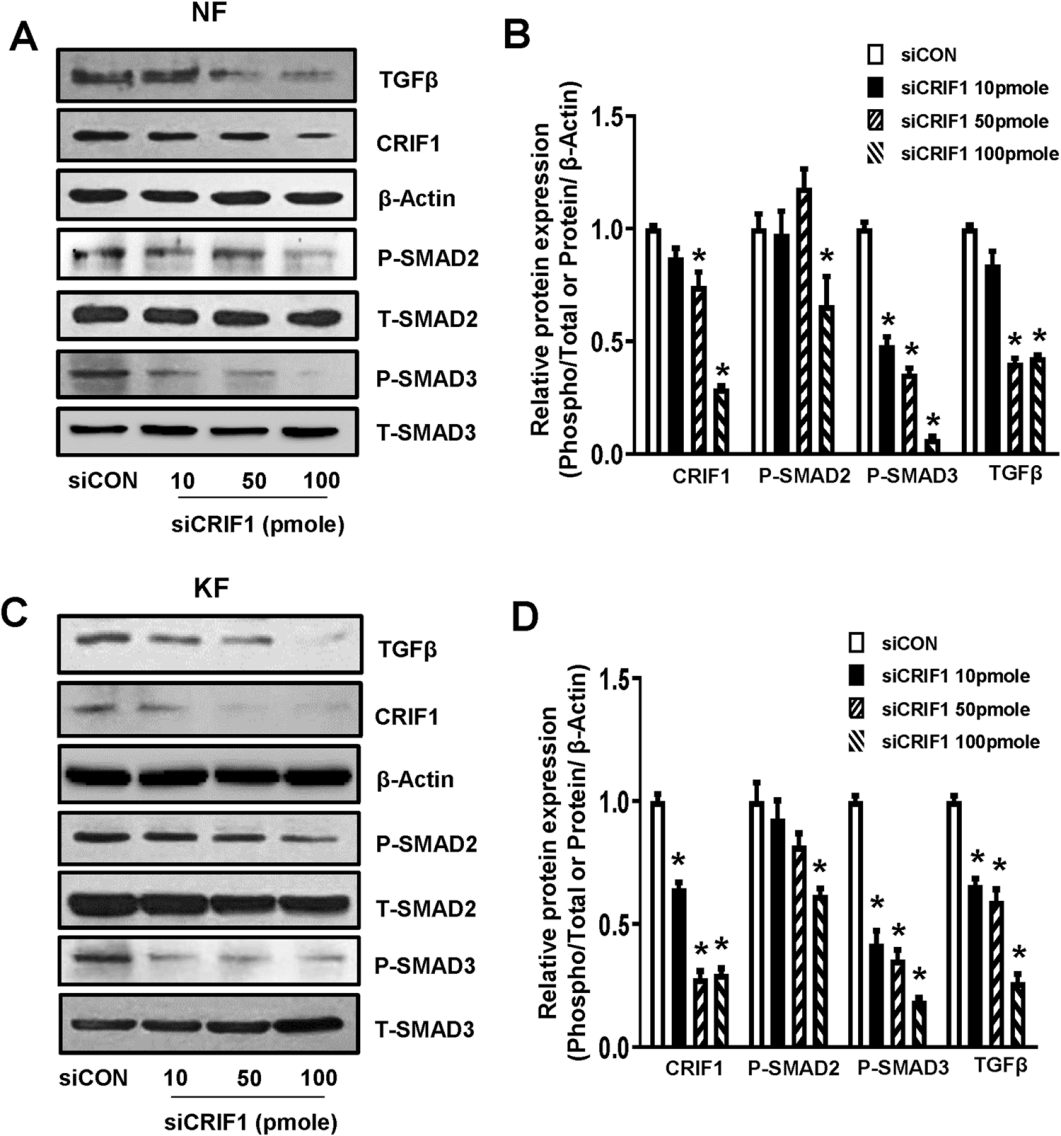
The TGF-β/Smad pathway is altered by CRIF1 downregulation in normal fibroblasts (NFs) and keloid fibroblasts (KFs). (**A**) NFs were transfected with CRIF1 siRNA in a dose-dependent manner for 48 h. After 48 h incubation, cells were harvested, lysed, and analyzed by western blot to assess differences in protein levels of TGF-β, P-Smad2, and P-Smad3; β-actin was used as the loading control. Full-length blots are presented in Supplementary Fig. S5a. (**B**) Protein density was calculated using ImageJ software. (**C**) KFs were transfected with CRIF1 siRNA in a dose-dependent manner for 48 h. After 48 h incubation, cells were harvested, lysed, and analyzed by western blot to assess differences in protein levels of TGF-β, P-Smad2, and P-Smad3; β-actin was used as the loading control. Full-length blots are presented in Supplementary Fig. S5b. (**D**) Protein density was calculated using ImageJ software. All data are presented as the mean ± SD of three independent experiments. *P < 0.05 compared to siCON.

**Figure 7:**
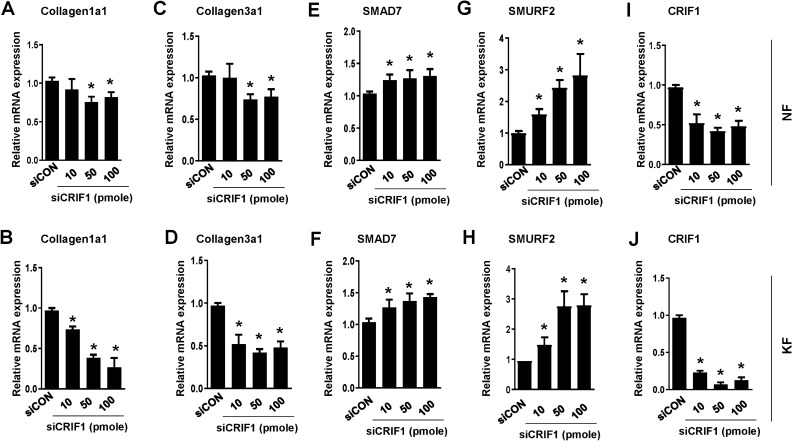
Expression of ECM components, Smad7 and SMURF2 mRNA levels are altered by CRIF1 downregulation in normal fibroblasts (NFs) and keloid fibroblasts (KFs). NFs were transfected with CRIF1 siRNA in a dose-dependent manner for 48 h. After 48 h incubation, mRNA expression levels of (**A**) COL1A1, (**C**) COL3A1 and (**E**) SMAD7, (**G**) SMURF2 and (**I**) CRIF1 were determined by qPCR. KFs were transfected with CRIF1 siRNA in a dose-dependent manner for 48 h. After 48 h incubation, mRNA expression levels of (**B**) COL1A1, (**D**) COL3A1 and (**F**) SMAD7, (**H**) SMURF2 and (**J**) CRIF1 were determined by qPCR. All data are presented as the mean ± SD of three independent experiments. *P < 0.05 compared to siCON.

Inhibition of the TGF-β/Smad signaling pathway markedly reduces keloid formation. Protein expression levels of TGF-β, phospho-Smad2, and phospho-Smad3 were measured by Western blotting after transfection of NFs (Fig. 6A,B) and KFs (Fig. 6C,D) with control or CRIF1 siRNA, to examine the changes in the TGF-β/Smad signaling pathway. TGF-β protein expression was significantly reduced in CRIF1-downregulated cells in both NFs and KFs. Similarly, phosphorylation of Smad2 and Smad3 was also decreased in response to CRIF1 downregulation. Subsequently, qPCR was performed to measure changes in the mRNA expression levels of the ECM components COL1A1 and COL3A1; the levels of COL1A1 in NFs (Fig. 7A) and KFs (Fig. 7B), and that of COL3A1 in NFs (Fig. 7C) and KFs (Fig. 7D) were all significantly decreased in response to CRIF1 downregulation.

### Smad7 and SMURF2 are increased by CRIF1 downregulation in normal and keloid fibroblasts

**Figure 8:**
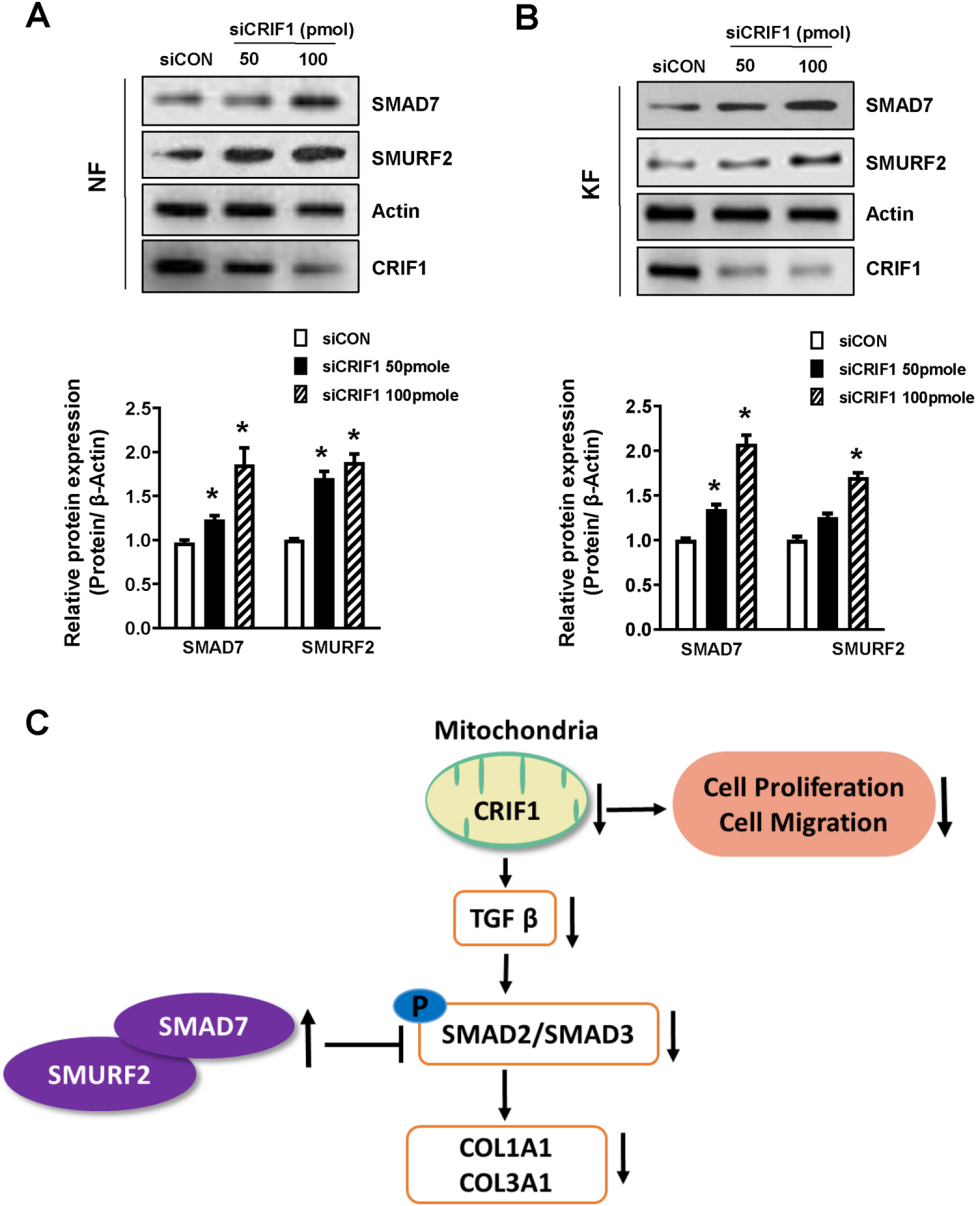
Smad7 and SMURF2 protein levels are altered by CRIF1 downregulation in normal fibroblasts (NFs) and keloid fibroblasts (KFs). (**A**) NFs were transfected with CRIF1 siRNA in a dose-dependent manner for 48 h. After 48 h incubation, cells were harvested, lysed, and analyzed by western blot to assess differences in protein levels of SMAD7 and SMURF2; β-actin was used as the loading control. Protein density was calculated using ImageJ software. Full-length blots are presented in Supplementary Fig. S6a. (**B**) KFs were transfected with CRIF1 siRNA in a dose-dependent manner for 48 h. After 48 h incubation, cells were harvested, lysed, and analyzed by western blot to assess differences in protein levels of SMAD7 and SMURF2; β-actin was used as the loading control. Protein density was calculated using ImageJ software. Full-length blots are presented in Supplementary Fig. S6b. All data are presented as the mean ± SD of three independent experiments. *P < 0.05 compared to siCON. (**C**) Schematic representation of CRIF1 downregulation induced changes in the TGF-β/Smad signaling pathway leading to the inhibition of invasive KFs.

Smad7 and SMURF2 are well known inhibitors of the Smad pathway. High expression of these proteins results in inhibition of the TGF-β signaling pathway, in turn causing decrease in cell proliferation and collagen deposition. As phosphorylation of Smad2 and Smad3 proteins was reduced under conditions of CRIF1 downregulation, we transfected NFs and KFs with either control or CRIF1 siRNA and examined the mRNA and protein levels of Smad7 and SMURF2. As shown in Fig. 7E,F, both Smad7 and SMURF2 mRNA expression levels were significantly increased by CRIF1 downregulation respectively in NFs as well as KFs (Fig. 7G,H). The protein expression of both Smad7 and SMURF2 was similarly found to be increased in NFs (Fig. 8A) and KFs (Fig. 8B). These results indicate that the increased expression of inhibitory proteins (Smad7 and SMURF2) may be beneficial for keloid growth.

## Discussion

Keloids arise as a result of excessive wound healing after skin damage, characterized by increases in cell proliferation, migration, inflammation, and collagen production, and excessive ECM synthesis^9^. In this study, we compared NFs and KFs under specific conditions. Previous reports have suggested that the anti-apoptotic effects of KFs in combination with increased proliferation and collagen deposition were higher than seen in NFs, with ~ 15% of genes being significantly upregulated in KFs^21,22^. This suggests that persistent differences in KFs are the main cause of keloid proliferation, making KFs a distinct cell type compared to NFs^23^. The fibroblasts that were extracted from keloids and normal skin tissues in this study were confirmed to have the characteristics of each tissue (Figs. 1, 2).

Mitochondria play an important role in energy metabolism via the production of ATP and other metabolites, serving as an essential source of the energy and biomolecules necessary to support tumor cell growth^24^. A large amount of reactive oxygen species (ROS) is produced as a byproduct of oxidative respiration in mitochondria, providing the energy necessary to fuel tumor cell proliferation. Vincent et al. showed that keloid metabolism is similar to that of tumor cells^15^. Keloids consumes more glucose than normal cells, and exhibits higher accumulation of lactate, although little is known regarding the effects of mitochondrial dysfunction in keloids. Previous studies have shown that deletion of CRIF1 results in poor synthesis of primary OXPHOS polypeptides and abnormal insertion into the mitochondrial inner membrane^13^. CRIF1 downregulation is a major factor underlying mitochondrial dysfunction in endothelial cells, with poor synthesis of OXPHOS subunits resulting in greater levels of mitochondrial dysfunction due to the accumulation of ROS^14^. CRIF1 has also been shown to function as an essential transcriptional coactivator, with Crif1^−^/^−^ embryos becoming inviable beyond embryonic day 6.5 due to extensive developmental arrest, accompanied by defective proliferation and extensive apoptosis^12^. In this study, we transfected siCRIF1 into NFs and KFs. This decrease in CRIF1 abundance led to decrease in cell proliferation, migration, and mRNA expressions of COL1A1 and COL3A1, all of which play an integral role in keloid formation (Figs. 3, 4, 5, 6, 7, 8). The data also reveals that downregulation of CRIF1 using siRNA has similar effects in both normal and keloid fibroblasts. Since cell proliferation, migration and TGF-β signaling are higher in KFs compared to NFs at the basal level, it can be said that reducing CRIF1 in keloid cells has a higher effect on the characteristics of these cells as compared to normal cells or it makes their phenotype resemble the normal cells.

TGF-β is among the most important cytokines driving the proliferation of fibroblasts and production of ECM. Smad proteins are a family of signaling proteins that participate in intracellular signal transduction of the TGF-β superfamily^25^. After TGF-β binds to its associated receptors, Smad2 and Smad3 are activated to form a trimer with Smad4. This trimer then migrates into the nucleus to regulate the transcription of target genes^26^. Smad7 is a key negative regulator of Smad, directly binding to TGF-β type I receptors resulting in the inhibition of Smad2 and Smad3 activation. Previous studies have shown that Smad7 is more specific for TGF-β signaling compared to other inhibitory Smads^27,28^. Importantly, Smad7 is downregulated in keloids, as well as other fibrotic tissues, such as the liver, lung, and kidney^29^. This low expression of Smad7 or other Smad-independent signaling pathways leads to the overproduction of collagen^30^. In our study, CRIF1 downregulation resulted in an increase in Smad7 and SMURF2 expression. Smad7 can bind tightly to the activated TGF-β-I receptor to prevent the phosphorylation of Smad. Taken together, these findings show that the biological effects of TGF-β under both normal and pathological conditions are tightly regulated by the coordination of Smad proteins in the TGF-β/Smad signaling pathway^28^. Our results suggest that ECM synthesis was affected by inhibition in phosphorylation of both Smad2 and Smad3 proteins, which are the transcription factors of COL1A1 and COL3A1 (Fig. 8C). The increase in Smad7 expression plays a key role in the decreased phosphorylation of Smad2 and Smad3, with some studies showing that COL1A1 and COL3A1 could be reduced by increasing the expression of Smad7^1,31^.

Therapeutic gene delivery is one of the effective treatments for certain diseases or genetic disorders at the cellular level^32,33^. Topical application of siRNA to treat skin disorders is of particular interest. For gene suppression in the skin, topical delivery of siRNA has various benefits such as direct access to the target site, reduced adverse effects which are sometimes associated with the systemic administration and visual monitoring of the affected region for any side effects^34^. There are various approaches described in previous studies for topical delivery of siRNAs^35–37^. The findings of the present study can be translated as a therapy to suppress keloid formation in the form of localized patch or topical delivery system such as an ointment, which could be applied at the keloid site and would suppress keloid formation by locally reducing CRIF1 expression.

A potential limitation of this study might be that keloids are specific to humans and there are some ethical limitations to conduct the studies in human patients. Moreover, no other animal species has been found to naturally develop a scar tissue, which is similar to that of human keloids. Therefore, the development of an animal model for detailed study of keloids has been extremely difficult. Many groups have attempted to generate animal models through two main approaches: (1) by inducing a comparable degree of fibrosis in an animal that does not normally develop it, or (2) by transplanting human-derived tissue or cells into the host animal for in vivo analysis^38,39^. For practically utilizing the findings of this study in the in vivo settings, the earlier approach of inducing fibrosis (keloid-like scar) in the CRIF1 knockout mice could be followed. However, either such scars are only maintained for a limited time period or they usually develop characteristics of hypertrophic scars. Secondly, transplantation of human keloid tissue/cell into the CRIF1 knockout mice could be done. Both these approaches can give researchers a reliable way to measure and study in detail about the keloid pathogenesis and developing a therapeutic strategy. Nevertheless, as both the methods have various challenges and limitations, similar attempts in the past have not been fully successful and there is still much to be done in the future. Finally, it can be concluded that CRIF1 downregulation impairs keloid function and suppresses their overgrowth via the transforming growth factor-β (TGF-β)/ Smad pathway.

## Materials and methods

### Primary keloid-derived and normal skin fibroblast cultures

This study was approved by the Institutional Review Board of Chungnam National University Hospital (IRB No. CNUH 2018-07-067) and was performed in accordance with the Helsinki Declaration of 1964 and its later amendments. Written informed consent was obtained from all patients. Samples were obtained from three patients (of both sexes) aged 18–45 years, with keloids of the earlobe. Surgical procedure involved the removal of keloid tissue along with the surrounding normal skin from each patient. The keloid tissue was used for isolating keloid fibroblasts and the surrounding normal skin was used for isolating normal fibroblasts. Cultures were established from tissue specimens processed within 6 h post-surgical excision. Biopsies were washed six times with distilled phosphate-buffered saline (DPBS) (Welgene, Gyeongsan, Republic of Korea) containing 1% antibiotics (Thermo Fisher Scientific, Waltham, MA, USA), and then incubated in Dulbecco’s modified Eagle’s medium (DMEM) (Welgene, Gyeongsan, Republic of Korea) for 30 min at 37 °C and 5% CO_2_ in a humidified atmosphere. Specimens were then transferred to a 60 mm^2^ cell culture plate (Corning Life Sciences, Corning, NY, USA) and incubated at room temperature for 30 min to allow the specimens to attach to its surface. DMEM containing 15% fetal bovine serum (FBS) (Atlas Biologicals, Fort Collins, CO, USA) and 1% antibiotics was then added to each plate. Plates were then returned to the 37 °C incubator and the culture medium was changed every 2 days.

### Cell culture and transfection

KFs and NFs were cultured in DMEM containing 10% FBS and 1% antibiotics, and incubated at 37 °C (5% CO_2_) in a humidified incubator. Culture medium was changed every 2–3 days. The cells were sub-cultured when confluence was above 80%. Primary cells were used at passages 3–7, at which point they were transfected with short interfering RNA (siRNA) for human CRIF1: sense: 5′-UGGAGGCCGAAGAACGCGAAUGGUA-3′ and antisense: 5′-UACCAUUCGCGUUCUUCGGCCUCCA-3′) (GenePharma, Shanghai, China) and negative control siRNA (GenePharma) using lipofectamine 2000 reagent (Invitrogen, Carlsbad, CA, USA) according to the manufacturer’s recommendations. The cells were incubated at 37 °C in a 5% CO2 incubator for 48 h for gene knockdown.

### Antibodies and western blotting

Mouse monoclonal anti-β-ACTIN, rabbit polyclonal anti-phospho Smad2, rabbit monoclonal anti-Smad2, rabbit monoclonal anti-phospho Smad3, rabbit polyclonal anti-Smad3, rabbit polyclonal anti-TGF-β, rabbit monoclonal anti-PCNA, and rabbit monoclonal anti-SMURF2 were obtained from Cell Signaling Technology (Beverly, MA, USA). Rabbit polyclonal anti-CRIF1 was obtained from Abcam (Cambridge, UK). Rabbit polyclonal anti-SMAD7 antibody was obtained from Invitrogen (Carlsbad, CA, USA). For Western blot, 15 μg of whole cell lysates was loaded and separated on 6–12% SDS-PAGE gels by electrophoresis, followed by incubation in the appropriate primary and secondary antibodies. For each western blot quantified, the experiment was repeated for a minimum of three times. Blots were imaged using a chemiluminescence assay kit (Miracle-Star Western Blot Detection System; Intron Biotechnology, Seongnam, Republic of Korea) and EZ-Western Lumi Femto (Daeil Lab Service, Seoul, Republic of Korea), and band densities were quantified on a Gel Doc 2000 Chemi Doc system using Quantity One software (Bio-Rad, Hercules, CA, USA). Values were normalized to β-actin (loading control).

### Cell proliferation

Cell proliferation in NFs and KFs was measured using the Cell Counting Kit-8 (CCK-8) (Dojindo, Rockville, MD, USA) according to the manufacturer’s instructions. Cells transfected with negative control or CRIF1 siRNA for 48 h were mixed with 50 μl CCK-8 solution and incubated for 1 h at 37 °C in a 5% CO2 humidified incubator, followed by measurement of absorbance at 450 nm. Cell proliferation was also measured using an ADAM-MC automatic cell counter (Digital Bio. Seoul, South Korea) that functions by using the propidium iodide (PI) staining method of dead cell staining. Cells were transfected with negative control or CRIF1 siRNA for 48 h and cell counting measurement was performed according to manufacturer’s instructions.

### Cell scratch assay

A cell scratch assay was used to assess cell migration. Cells were transfected with negative control or CRIF1 siRNA in 6-well tissue culture plates for 24 h, after which a sterile 1 ml pipette tip was used to detach the cells from the monolayer across the center of the well. Floating cells were flushed out by gently rinsing with PBS and replaced with serum-free medium (to rule out cell proliferation as the cause of wound closure) followed by incubation for another 24 h. The total incubation time post transfection was therefore 48 h. Cell movement was monitored microscopically. Photographs were taken immediately and at 24 h after scratching. The migration capacity of cells was expressed as a percentage of wound coverage: relative migrated distance = (A_0h_ − A_24h_)/A_0h_ × 100%, where A_0h_ and A_24h_ represent the wound area measured immediately and 24 h after scratching, respectively. The wound area was quantitatively evaluated using ImageJ software (NIH, Bethesda, MD, USA).

### Transwell assay

Transwell assay was employed to detect cell migration. Cells were transfected with negative control or CRIF1 siRNA in 6-well tissue culture plates for 24 h, followed by transfer of 5 × 10^5^/ml cells in the upper transwell chamber (24-well plate; Corning, New York, USA) and culture with FBS free medium. Complete growth medium with 10% FBS was added to the lower chamber and incubated for another 24 h. Then, cells on the upper side (nonmigrating cells) were removed and migrated cells on the lower face were washed with PBS, fixed with 4% paraformaldehyde, stained with DAPI and counted on 5 random high-power fields (× 200 magnification) under a microscope and averaged.

### Mitomycin C treatment

To rule out cell proliferation as the cause of migration in transwell experiments; a cell proliferation inhibitor Mitomycin C (MMC) was used. 4 h after transfection of Normal or Keloid fibroblasts with CRIF1 siRNA, MMC (Sigma M4287) was treated at a dose of 0.04 mg/ml for 5 min in serum free media. After flushing out MMC, cells were replaced with fresh media.

### Real-time polymerase chain reaction (PCR)

Total RNA was isolated from cells using TRIzol reagent (Thermo Fisher Scientific). Complementary DNA (cDNA) was generated using an RT premix kit (iNtRON Biotechnology, Seongnam, South Korea). Relative RNA expression levels were determined by PCR using a SYBR qPCR premix (Enzynomics, Daejeon, Republic of Korea). The primer sequences were as follows: GAPDH: 5′-GGAGCGAGATCCCTCCAAAAT-3′ (forward) and 5′-GGCTGTTGTCATACTTCTCATGG-3′ (reverse); COL1A1: 5′-GAGGGCCA AGACGAAGACATC-3′ (forward) and 5′-CAGATCACGTCATCGCACAAC-3′ (reverse); COL3A1: 5′-GGAGCTGGCTACTTCTCGC-3′ (forward) and 5′-GGGAACATCCTCCTTCAACAG-3′ (reverse); CRIF1: 5′-GCACGCAGCCTACTAGGTG-3′ (forward) and 5′-CGAACTGCTTAGCCGCGTA-3′ (reverse); Smad7: 5′-GGACAGCTCAATTCGGACAAC-5′ (forward) and reverse-5′-GTACACCCACACACC ATCCAC-3′; Smurf2: 5′-TATGCAAACTCGGGCCAAATG-3′ (forward) and 5′-CCTGTGCCTATTCGGTCTCTG-3′ (reverse). The PCR cycling conditions were as follows: 5 min at 95 °C, 40 cycles of 30 s at 95 °C, 30 s at 60 °C and 30 s at 72 °C, followed by 5 min at 72 °C. GAPDH was used as an internal control. Results were interpreted by the relative quantity method (ΔΔCt).

### Statistical analysis

All experiments were performed at least three times for each donor. Data are presented as means ± SD. Statistical significance was determined using Student’s t-test, with *p*-values < 0.05 considered significant.

## Supplementary Information

Supplementary Information.

## Data Availability

The datasets generated during the current study are available from the corresponding author on reasonable request.
