# Supplementary material for: Downregulation of CR6-interacting factor 1 suppresses keloid fibroblast growth via the TGF-β/Smad signaling pathway
Source: Sci Rep. 2021 Jan 12;11:500. doi: 10.1038/s41598-020-79785-y (PMC7804403; doi:10.1038/s41598-020-79785-y)
Supplement: Supplementary file 1 — Supplementary Information. [file 41598_2020_79785_MOESM1_ESM.pptx]

## Slide 1
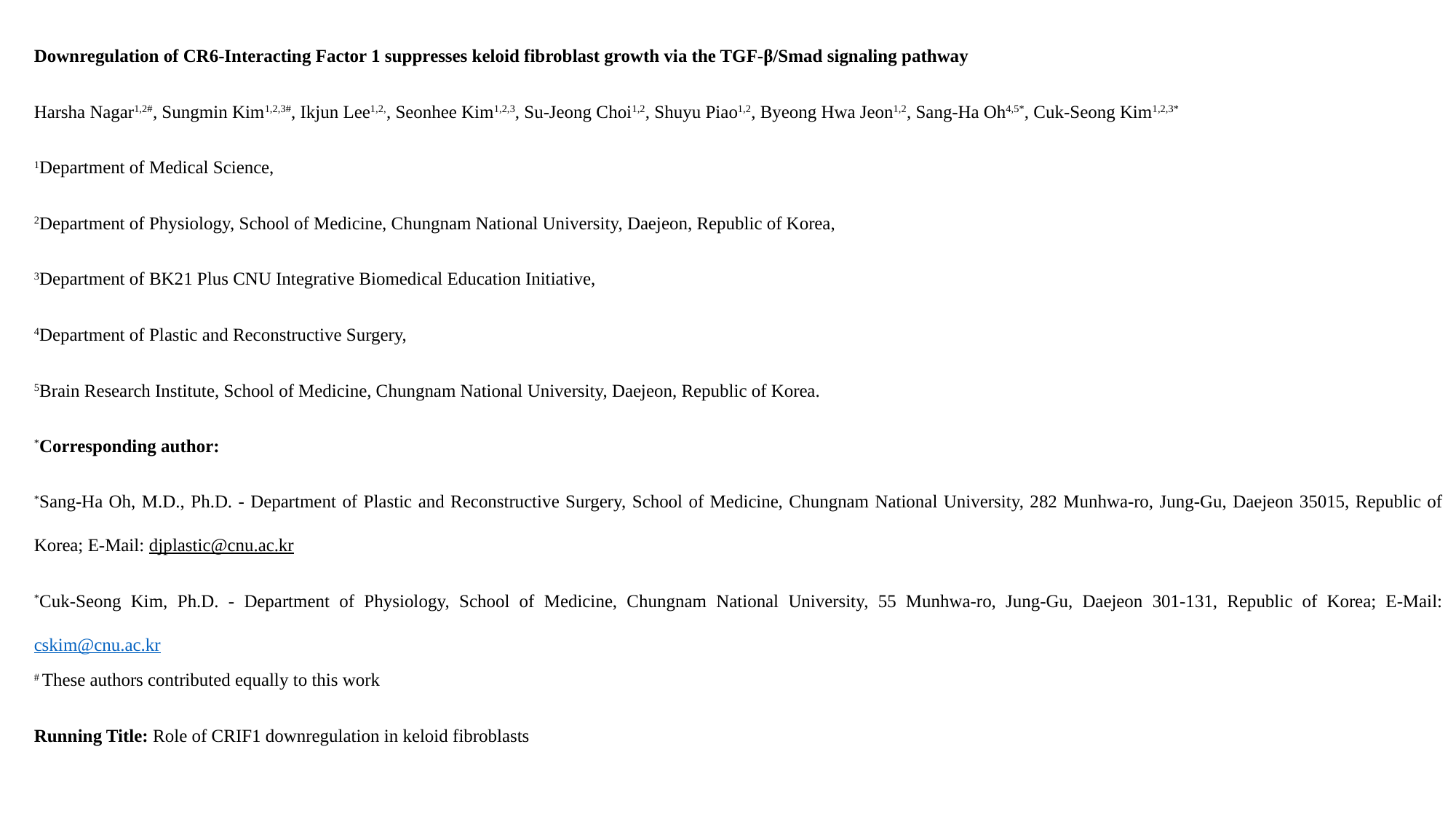

Downregulation of CR6-Interacting Factor 1 suppresses keloid fibroblast growth via the TGF-β/Smad signaling pathway
Harsha Nagar1,2#, Sungmin Kim1,2,3#, Ikjun Lee1,2,, Seonhee Kim1,2,3, Su-Jeong Choi1,2, Shuyu Piao1,2, Byeong Hwa Jeon1,2, Sang-Ha Oh4,5*, Cuk-Seong Kim1,2,3*
1Department of Medical Science,
2Department of Physiology, School of Medicine, Chungnam National University, Daejeon, Republic of Korea,
3Department of BK21 Plus CNU Integrative Biomedical Education Initiative,
4Department of Plastic and Reconstructive Surgery,
5Brain Research Institute, School of Medicine, Chungnam National University, Daejeon, Republic of Korea.
*Corresponding author:
*Sang-Ha Oh, M.D., Ph.D. - Department of Plastic and Reconstructive Surgery, School of Medicine, Chungnam National University, 282 Munhwa-ro, Jung-Gu, Daejeon 35015, Republic of Korea; E-Mail: djplastic@cnu.ac.kr
*Cuk-Seong Kim, Ph.D. - Department of Physiology, School of Medicine, Chungnam National University, 55 Munhwa-ro, Jung-Gu, Daejeon 301-131, Republic of Korea; E-Mail: cskim@cnu.ac.kr
# These authors contributed equally to this work
Running Title: Role of CRIF1 downregulation in keloid fibroblasts

## Slide 2
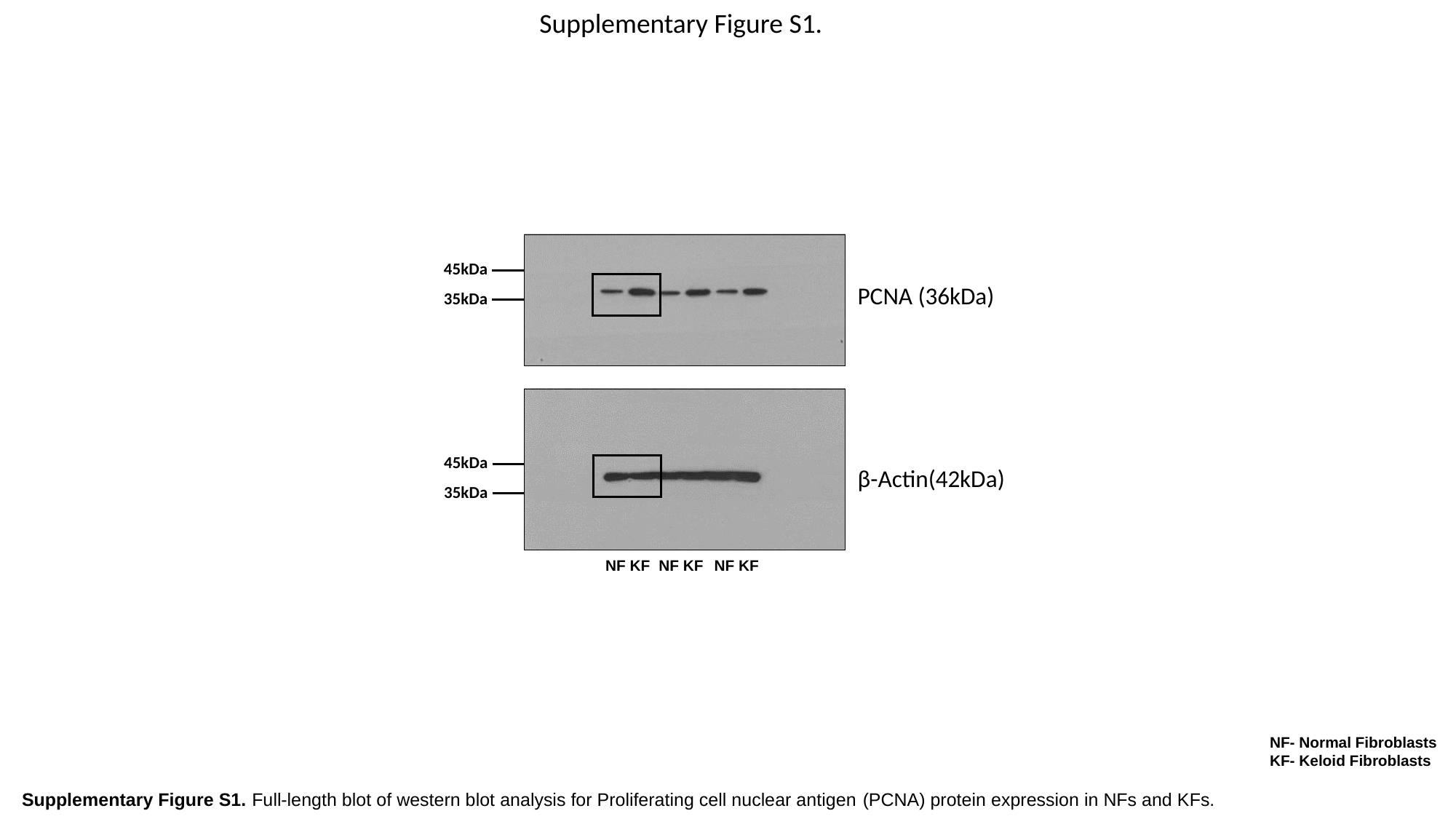

Supplementary Figure S1.
45kDa
PCNA (36kDa)
35kDa
45kDa
β-Actin(42kDa)
35kDa
NF KF
NF KF
NF KF
NF- Normal Fibroblasts
KF- Keloid Fibroblasts
Supplementary Figure S1. Full-length blot of western blot analysis for Proliferating cell nuclear antigen (PCNA) protein expression in NFs and KFs.

## Slide 3
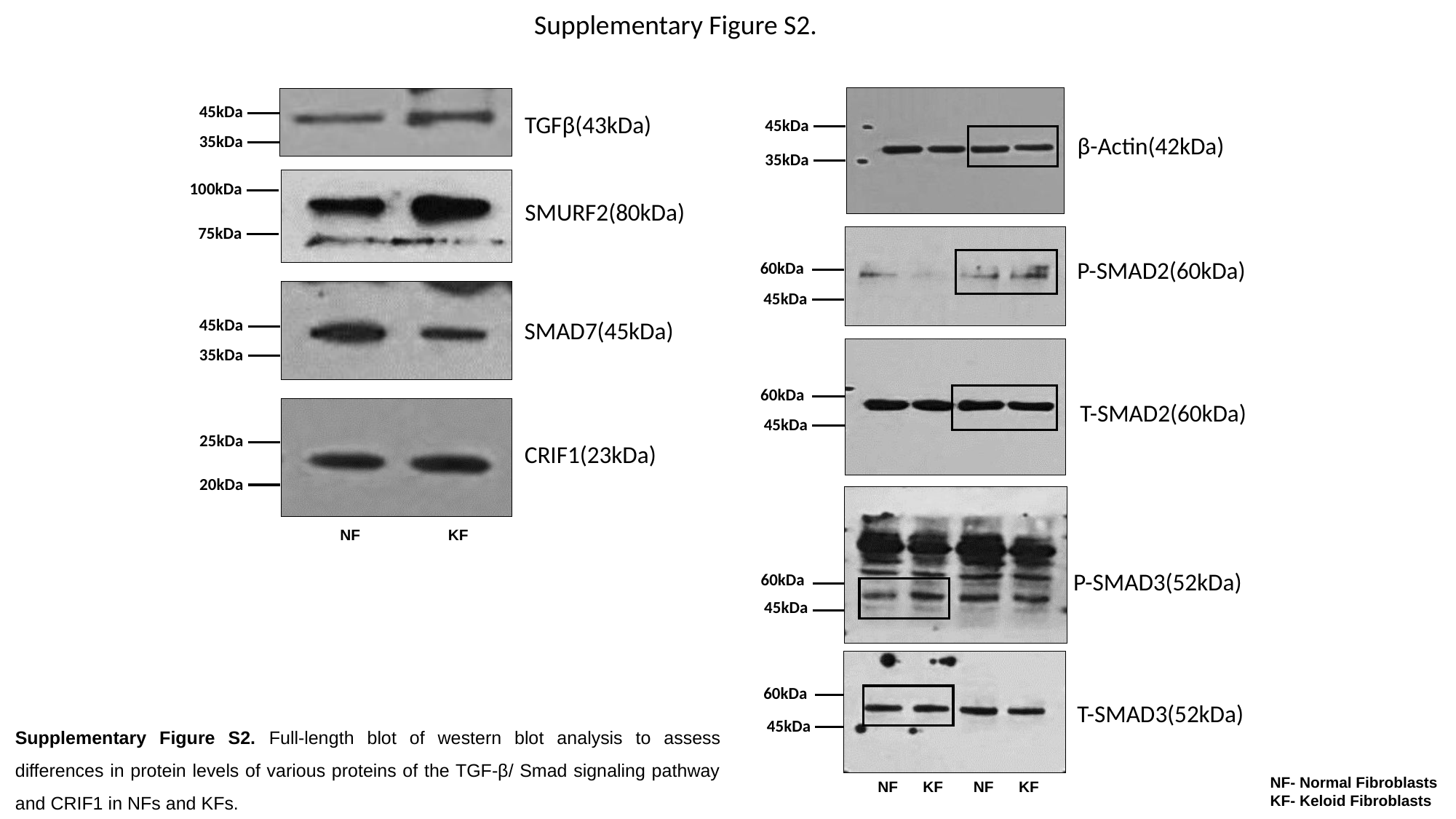

Supplementary Figure S2.
45kDa
TGFβ(43kDa)
45kDa
35kDa
β-Actin(42kDa)
35kDa
100kDa
SMURF2(80kDa)
75kDa
P-SMAD2(60kDa)
60kDa
45kDa
45kDa
SMAD7(45kDa)
35kDa
60kDa
T-SMAD2(60kDa)
45kDa
25kDa
CRIF1(23kDa)
20kDa
 NF KF
P-SMAD3(52kDa)
60kDa
45kDa
60kDa
T-SMAD3(52kDa)
Supplementary Figure S2. Full-length blot of western blot analysis to assess differences in protein levels of various proteins of the TGF-β/ Smad signaling pathway and CRIF1 in NFs and KFs.
45kDa
NF- Normal Fibroblasts
KF- Keloid Fibroblasts
NF KF
NF KF

## Slide 4
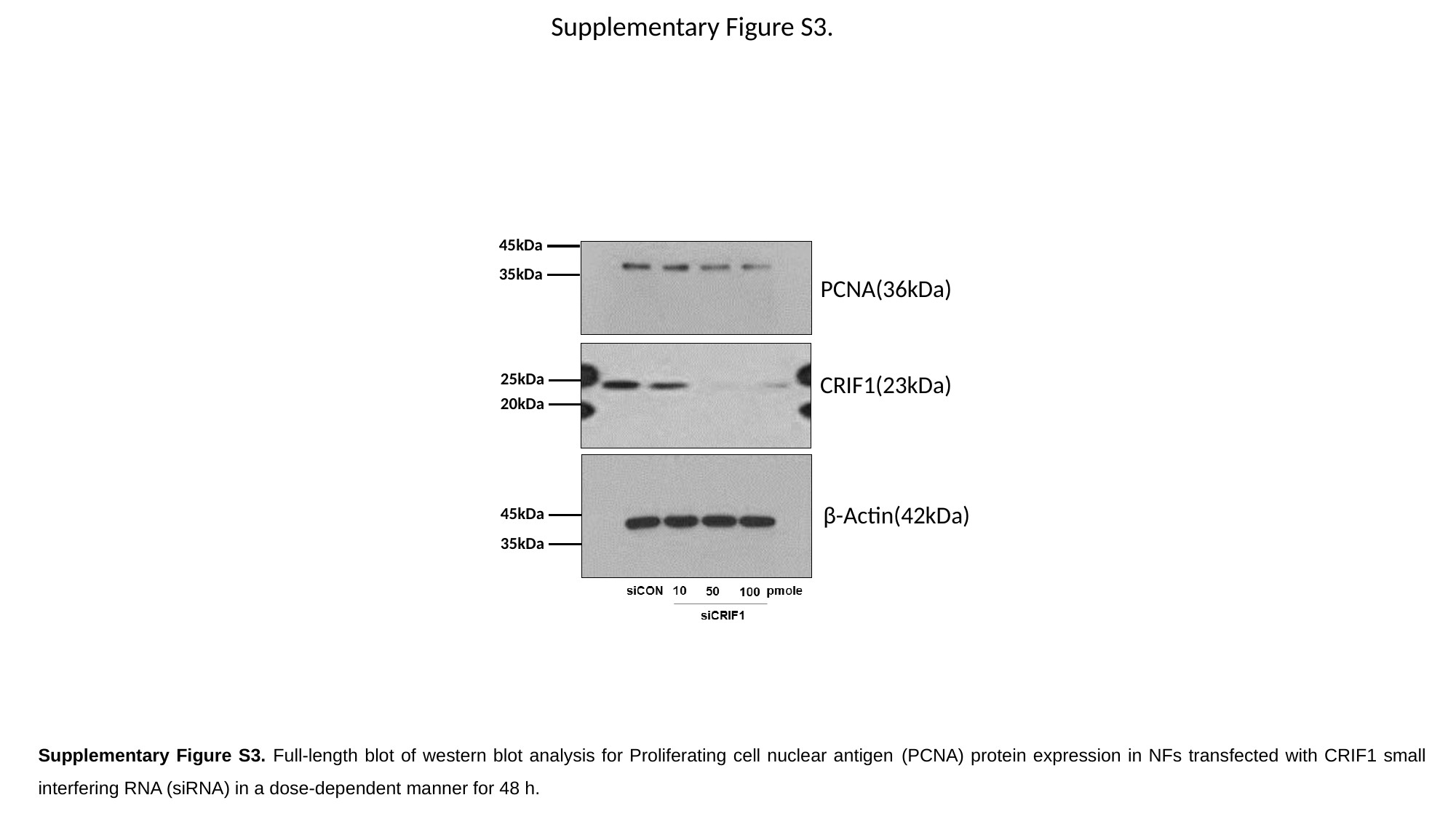

Supplementary Figure S3.
45kDa
35kDa
PCNA(36kDa)
25kDa
CRIF1(23kDa)
20kDa
β-Actin(42kDa)
45kDa
35kDa
Supplementary Figure S3. Full-length blot of western blot analysis for Proliferating cell nuclear antigen (PCNA) protein expression in NFs transfected with CRIF1 small interfering RNA (siRNA) in a dose-dependent manner for 48 h.

## Slide 5
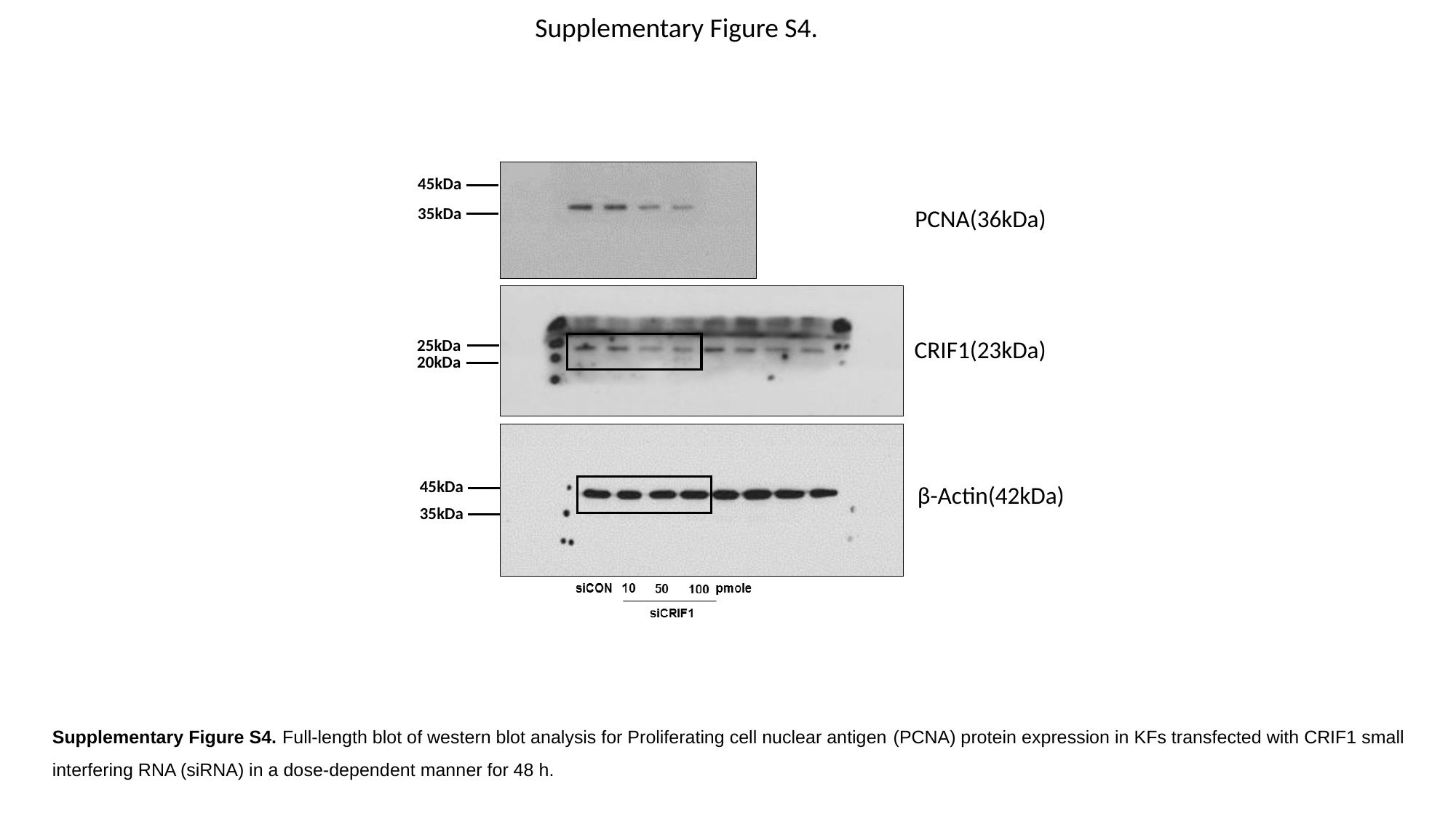

Supplementary Figure S4.
45kDa
35kDa
PCNA(36kDa)
25kDa
CRIF1(23kDa)
20kDa
45kDa
β-Actin(42kDa)
35kDa
Supplementary Figure S4. Full-length blot of western blot analysis for Proliferating cell nuclear antigen (PCNA) protein expression in KFs transfected with CRIF1 small interfering RNA (siRNA) in a dose-dependent manner for 48 h.

## Slide 6
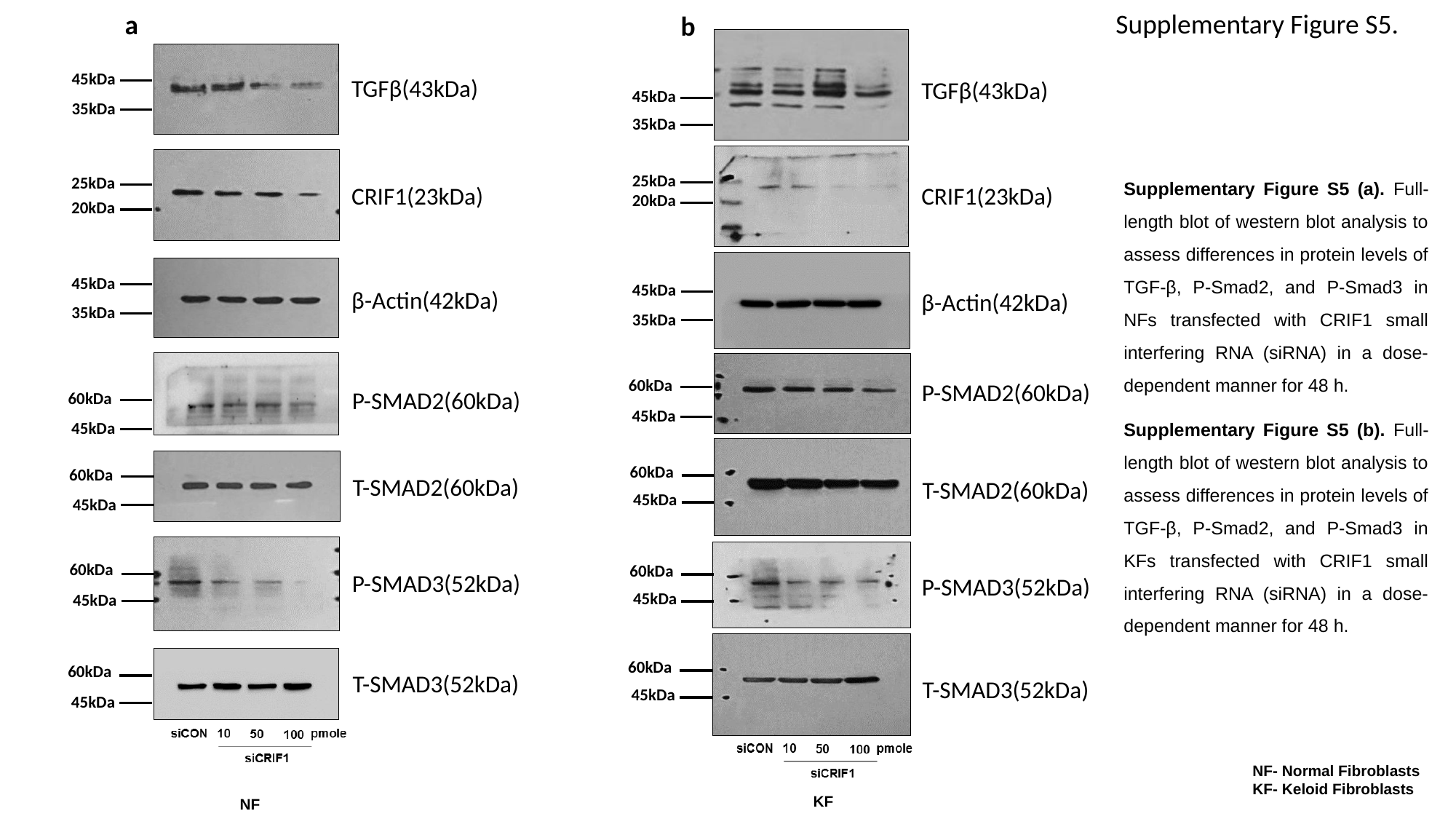

Supplementary Figure S5.
a
b
45kDa
TGFβ(43kDa)
TGFβ(43kDa)
45kDa
35kDa
35kDa
Supplementary Figure S5 (a). Full-length blot of western blot analysis to assess differences in protein levels of TGF-β, P-Smad2, and P-Smad3 in NFs transfected with CRIF1 small interfering RNA (siRNA) in a dose-dependent manner for 48 h.
Supplementary Figure S5 (b). Full-length blot of western blot analysis to assess differences in protein levels of TGF-β, P-Smad2, and P-Smad3 in KFs transfected with CRIF1 small interfering RNA (siRNA) in a dose-dependent manner for 48 h.
25kDa
25kDa
CRIF1(23kDa)
CRIF1(23kDa)
20kDa
20kDa
45kDa
45kDa
β-Actin(42kDa)
β-Actin(42kDa)
35kDa
35kDa
60kDa
P-SMAD2(60kDa)
P-SMAD2(60kDa)
60kDa
45kDa
45kDa
60kDa
60kDa
T-SMAD2(60kDa)
T-SMAD2(60kDa)
45kDa
45kDa
60kDa
60kDa
P-SMAD3(52kDa)
P-SMAD3(52kDa)
45kDa
45kDa
60kDa
60kDa
T-SMAD3(52kDa)
T-SMAD3(52kDa)
45kDa
45kDa
NF- Normal Fibroblasts
KF- Keloid Fibroblasts
KF
NF

## Slide 7
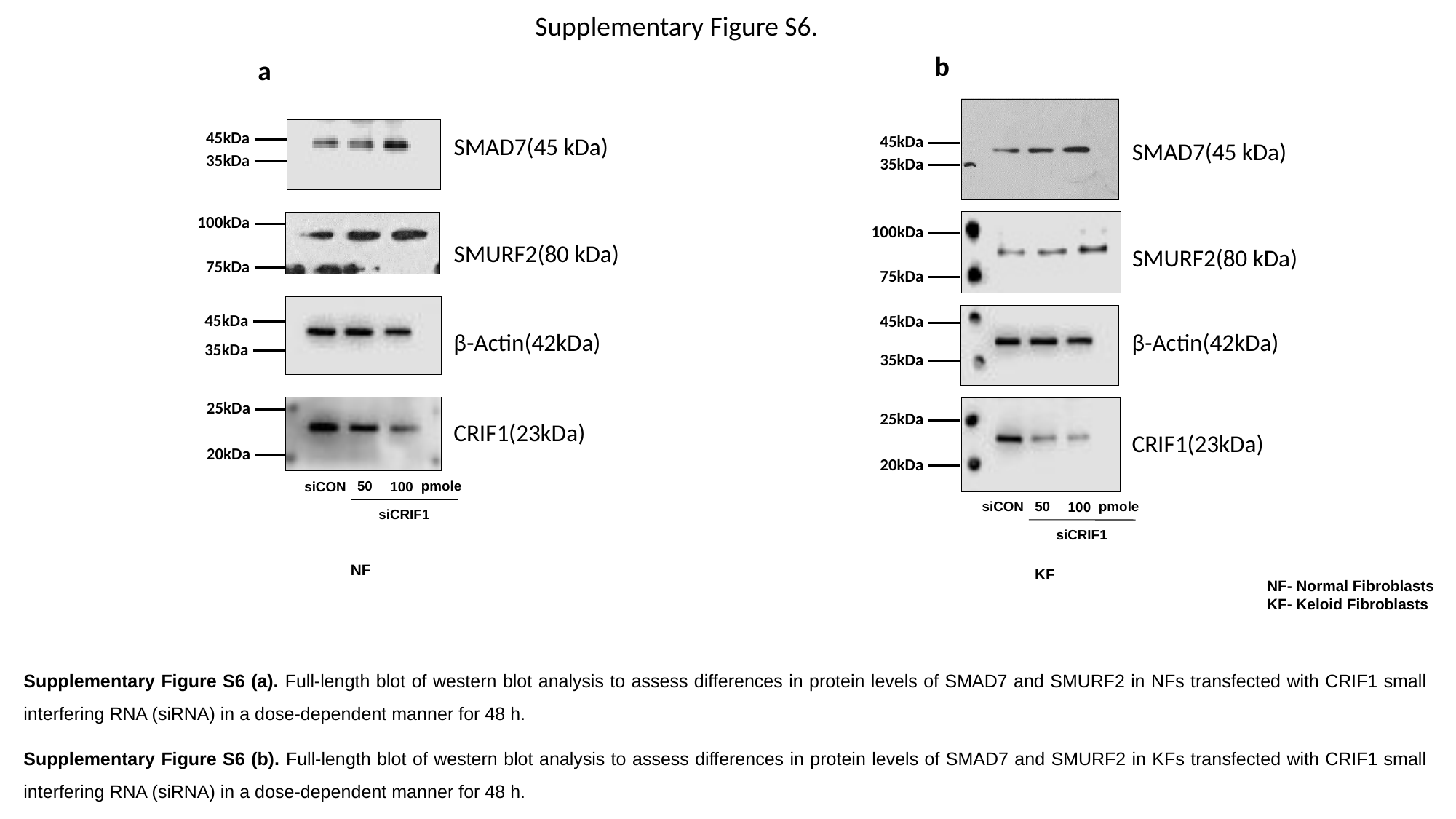

Supplementary Figure S6.
b
a
45kDa
SMAD7(45 kDa)
45kDa
SMAD7(45 kDa)
35kDa
35kDa
100kDa
100kDa
SMURF2(80 kDa)
SMURF2(80 kDa)
75kDa
75kDa
45kDa
45kDa
β-Actin(42kDa)
β-Actin(42kDa)
35kDa
35kDa
25kDa
25kDa
CRIF1(23kDa)
CRIF1(23kDa)
20kDa
20kDa
pmole
50
siCON
100
pmole
50
siCON
100
siCRIF1
siCRIF1
NF
KF
NF- Normal Fibroblasts
KF- Keloid Fibroblasts
Supplementary Figure S6 (a). Full-length blot of western blot analysis to assess differences in protein levels of SMAD7 and SMURF2 in NFs transfected with CRIF1 small interfering RNA (siRNA) in a dose-dependent manner for 48 h.
Supplementary Figure S6 (b). Full-length blot of western blot analysis to assess differences in protein levels of SMAD7 and SMURF2 in KFs transfected with CRIF1 small interfering RNA (siRNA) in a dose-dependent manner for 48 h.
